# 

**Published:** 1903-12

**Authors:** 


### SOUTHERN SURGICAL AND GYNECOLOGICAL ASSOCIATION MEETING.

The Southern Surgical and Gynecological Association meets in Atlanta at the Piedmont Hotel, December 15, 16 and 17.

Preparations are being made for the entertainment of the members under the chairmanship of Dr. Willis Westmoreland, and there is every prospect of a full attendance. The following is the preliminary program:

1. Presidential Address, by Dr. J. W. Bovee, Washington.
2. Memorial Address on Dr. W. E. B. Davis, by Dr. Richard Douglas, Nashville.

3. A Study of the Prostate and Treatment of Obstructive Hypertrophy, by Dr. G. Wiley Broome, St. Louis.

4. Surgical Intervention in Congenital Cystic Kidneys, by Dr. W. G. McDonald, Albany.

5. The Significance and Management of Paralysis of the Bowel, by Dr. George S. Brown, Birmingham.

6. The Pneumatic Rubber Suit as a Means of Controlling the Blood Pressure, with a Demonstration of the Apparatus, by Dr. George W. Crile, Cleveland.

7. (a) Edematous Encephalitis: A Study of the Conditions Found in Operating for Cerebral Epilepsy and Allied Conditions. (b) A new Device and Method for Intestinal Anastomosis, by Dr. W. P. Carr, Washington.

8. The Recognition and Treatment of Congenital Misplacements of the Hip in Infancy, by Dr. A. H. Freiberg, Cincinnati.

9. Fracture—Dislocation of the Spine, by Dr. J. Shelton Horsley, Richmond.

10. Neoplasms, Wherever Situated, Should, if Possible, be Removed, Whatever Their Apparent Nature, by Dr. M. H. Richardson, Boston.

11. Operative Treatment of Cancer of the Liver with Report of Case, by Dr. Joseph Ransohoff, Cincinnati.

12. Intestinal Cancer, by Dr. J. Garland Sherrill, Louisville.

13. The Early Diagnosis of Cancer of the Uterus, by Dr. F. F. Simpson, Pittsburg.

14. Case of Carcinoma of the Rectum with Excision by Combined Perineal and Abdominal Route, with Exhibition of Specimen, by Dr. J. Horace Whitacre, Cincinnati.

15. Differential Diagnosis of Epithelioma and Syphilis of the Vulva, by Dr. Barton Cooke Hirst, Philadelphia.

16. Spurious Dysmenorrhea: An Appeal for a More Rational Treatment, by Dr. Henry T. Byford, Chicago.

17. Tuberculosis of the Uterus, by Dr. Charles M. Rees, Charleston.

18. Surgery of Urinary Tuberculosis in Women, by Dr. Guy L. Hunner, Baltimore.

19. Surgery without Ligatures, by Dr. A. J. Downes, Philadelphia.

20. Further Experience with the Use of the Downes Electro-Thermic Clamp in the Treatment of Cancer of the Uterus, by Dr. Charles P. Noble, Philadelphia.

21. Should We not Do Myomectomy rather than Hysterectomy, if Possible, While Operating for Uterine Fibromyomata, and Shall We not Leave

Healthy Ovaries when We Do a Hysterectomy? by Dr. C. C. Frederick, Buffalo.

22. Ectopic Gestation at Term, with Illustrations, by Dr. Charles A. L. Reed, Cincinnati.

23. On the Practical Progress we have made in Pelvic and Abdominal Surgery, by Dr. Joseph Price, Philadelphia.

24. Pus Collections in the Pelvis, by Dr. C. U. Chivigny, New Orleans.

25. Technic of Operations by the Vaginal Method, by Dr. J. Riddle Goffe, New York.

26. (a) Tubercular Ostitis of the Knee-Joint. (b) An Exhibition of a Plaster-of-Paris Bandage Roller, by Dr. A. R. Sands, Washington.

27. Arthroplasty, by Dr. J. B. Murphy, Chicago.

28. Seven Cases of Amputation of the Hip-joint for Sarcoma without Mortality, by Dr. W. B. Coley, New York.

29. Excision of Psoas Magnus Muscle for Sarcoma, by Dr. George H. Noble, Atlanta.

30. Appendicitis, by Dr. J. M. Baldy, Philadelphia.

31. Post-Peritoneal Infection Following Appendicitis, by Dr. I. S. Stone, Washington.

32. Notes on the Short Incision in Appendicitis Work, by Dr. Robert T. Morris, New York.

33. The Surgical Physiology of the Lymphatic System, by Dr. C. H. Mayo, Rochester.

34. Septic Thrombosis of the Femoral Vein following Influenza; Report of Case, by Dr. Dyer F. Talley, Birmingham.

35. Gun-Shot Wounds of the Lung, by Dr. W. C. Borden, U. S. Army.

36. Five Recent Successful Laparotomies for Gun-Shot Wounds of Abdominal Viscera, including one with Twenty-eight Perforations of Intestine and Mesentery, by Dr. Robert W. Johnson, Baltimore.

37. Atypical Cases: (1) Diphtheria of the Genitalia during the Puerperium. (2) Rupture of the Small Intestine. (3) Strangulated Inguinal Hernia Containing the Uterus and Fallopian Tubes in an Infant, by Dr. J. W. Long, Greensboro.

38. Gall Stones, by Dr. Mack Rogers, Birmingham.

39. Vaginal Cystotomy for Stone in the Bladder, by Dr. Charles R. Robins, Richmond.

40. Remarks on the Treatment of Abortion, with Prophylaxis in Special Cases, by Dr. Thad. A. Reamy, Cincinnati.

41. Free Growths in the Tunica Vaginalis, by Dr. E. A. Balloch, Washington.

42. Complications to be Met in the Surgical Treatment of the Testicle, by Dr. J. McF. Gaston, Jr., Atlanta.

43. Fractures at the Elbow, by Dr. J. B. Murfree, Murfreesboro.

44. Inguinal Hernia, by Dr. W. F. Westmoreland, Atlanta.

45. The Regulation of the Length of Exposure and the Distance from the Tube in X-Ray Therapy, by Dr. Ennion G. Williams, Richmond.

46. The Surgical Treatment of Acute Pancreatitis, by Dr. W. D. Haggard, Nashville.

**This Journal and Atlanta Semi-weekly Journal one year for $1.25 cash in advance. No checks. Strictly to new subscribers.**

